# Toward Microbial Recycling and Upcycling of Plastics: Prospects and Challenges

**DOI:** 10.3389/fmicb.2022.821629

**Published:** 2022-03-23

**Authors:** Jo-Anne Verschoor, Hadiastri Kusumawardhani, Arthur F. J. Ram, Johannes H. de Winde

**Affiliations:** ^1^Molecular Biotechnology, Institute of Biology, Leiden University, Leiden, Netherlands; ^2^Department of Fundamental Microbiology, University of Lausanne, Lausanne, Switzerland

**Keywords:** plastics, enzymes, biorecycling, biodegradation, comprehensive workflow

## Abstract

Annually, 400 Mt of plastics are produced of which roughly 40% is discarded within a year. Current plastic waste management approaches focus on applying physical, thermal, and chemical treatments of plastic polymers. However, these methods have severe limitations leading to the loss of valuable materials and resources. Another major drawback is the rapid accumulation of plastics into the environment causing one of the biggest environmental threats of the twenty-first century. Therefore, to complement current plastic management approaches novel routes toward plastic degradation and upcycling need to be developed. Enzymatic degradation and conversion of plastics present a promising approach toward sustainable recycling of plastics and plastics building blocks. However, the quest for novel enzymes that efficiently operate in cost-effective, large-scale plastics degradation poses many challenges. To date, a wide range of experimental set-ups has been reported, in many cases lacking a detailed investigation of microbial species exhibiting plastics degrading properties as well as of their corresponding plastics degrading enzymes. The apparent lack of consistent approaches compromises the necessary discovery of a wide range of novel enzymes. In this review, we discuss prospects and possibilities for efficient enzymatic degradation, recycling, and upcycling of plastics, in correlation with their wide diversity and broad utilization. Current methods for the identification and optimization of plastics degrading enzymes are compared and discussed. We present a framework for a standardized workflow, allowing transparent discovery and optimization of novel enzymes for efficient and sustainable plastics degradation in the future.

## Introduction—The World of Plastics

Plastics are man-made polymers that are used for many applications. Their flexibility, strength and erosion resistance allow plastics to be suitable material for a broad spectrum of applications (Wang et al., 2018). Of the almost 400 Mt of plastics produced, 40 percent is used in single-use applications, leading to a significant amount of waste (PlasticEurope-Association of Plastics Manufacturers, 2020). Current waste management systems mostly consist of accumulation in landfills, incineration for energy recovery, and recycling. Although the fraction of plastic that is being recycled is increasing, the vast majority ends up in the incinerator or in landfills polluting the environment, rather than being reused (Geyer et al., 2017). Especially in Asia millions of metric tons of plastic waste are managed poorly resulting in a high likelihood of leakage into the environment (d’Ambrières, 2019).

Landfilling, careless dumping and other sources of leakage such as the release of microfibres into the environment, causes the accumulation of plastics in both terrestrial and marine ecosystems (Henry et al., 2019). The leakage of plastics into the environment has been shown to have several negative effects on flora and fauna. Environmentally, plastic pollution has a plethora of effects. The presence of plastic films in soil has been shown to decrease crop yield by 3% meaning that this could have significant effects on food security (Zhang et al., 2020). The fragmentation of larger plastic products, when exposed to abiotic factors, into so-called nano (<100 nm) and/or microplastics (<5 mm) is also a risk that needs to be considered (Science Advice for Policy by European Academies [SAPEA], 2019; Chamas et al., 2020). Even though the SAPEA report from 2019 states that micro and nano plastics do not endanger human health and the environment, the evidence seemed limited at that time (Science Advice for Policy by European Academies [SAPEA], 2019). Recently, several studies and reviews regarding the effects of nano and microplastics on the environment have been published and describe that there is a negative effect of these particles in the environment (Shen et al., 2019; Iqbal et al., 2020; Selonen et al., 2020; Shiu et al., 2020; Tiwari et al., 2020; Guerrera et al., 2021; Kumar et al., 2021; Wang et al., 2022; Zhang et al., 2022). The effects of these particles existent from neurotoxicity to oxidative stress and even lethality (Wang et al., 2021). The further degradation of monomers and oligomers of plastics can also result in negative health effects. Exposure of pregnant mice to bisphenol A and S resulted in the disruption of their placentas (Mao et al., 2020). Finally, the release of harmful volatile organic compounds upon photo-degradation of plastics has been neglected until recently meaning that there might be more aspects of plastic degradation threatening our ecosystems (Lomonaco et al., 2020).

The results so far show that plastic pollution harms our planet, meaning that we should aim to decrease plastic pollution. According to a recent model (Lau et al., 2020), several approaches need to be taken in parallel to decrease the rapid accumulation of plastics in the environment: (1) plastic consumption needs to be reduced by limiting single-use plastic products; (2) reuse should be stimulated; (3) waste collection and recycling should be increased; (4) landfilling should eventually be eliminated.

To increase recycling, more efficient collecting, sorting and recycling methods should be developed. A promising and novel recycling approach is biorecycling, which relies on the enzymatic degradation of plastics. After degradation, the monomers are extracted and used to manufacture the original plastic known as recycling or be used as building blocks for other compounds with a higher value (upcycling), without loss of quality.

Sorting waste materials after collection is an essential step in the implementation of recycling as the main method for domestic plastic waste management. As described by Hahladakis and Iacovidou (2019), the lack of efficient sorting may cause unwanted blending of polymers, ultimately yielding new polymeric materials with unwanted characteristics. In addition, accidental mixing of biobased plastics with petrochemical-based plastics compromises the recyclability of the end products (Hahladakis and Iacovidou, 2019). Currently, automated sorting is being employed based on material or color focused separation. Both methods have clear limitations and contamination cannot be avoided (Hahladakis and Iacovidou, 2019). Automated sorting of plastics using near-infrared spectroscopy may separate various household plastics with an accuracy of 99% (Wu et al., 2020). However, this method still needs further improvement since it is not suitable for dark-colored plastics. Moreover, interference and effects of surface coating and surface contamination still need further characterization (Wu et al., 2020).

Despite the limitations in plastic sorting, various recycling methods are in use. For large scale solid waste, mechanical recycling is the preferred method. In this method, all organic residues are washed off which is followed by shredding, melting, and remolding of the polymeric material. Since household waste consists of a wide variety of polymers, sometimes contaminated with coloring, other plastics or metals, the quality of the reprocessed matter is relatively low (Eriksen et al., 2018; Shahid Kashif et al., 2021). To overcome this low quality, mixing with virgin plastic is required to achieve the desirable characteristics for industrial use (Garcia and Robertson, 2017). Another approach is chemical recycling, yielding higher quality products but having significantly higher costs. Examples of chemical recycling are the lysis of glycols and methanol (Grigore, 2017). However, the chemical structure and diversity as well as the use of various plastics mixtures, compromise the efficiency of mechanical recycling (Garcia and Robertson, 2017), and are the main cause for the fact that most of the solid waste is not being recycled. Since energy recovery and mechanical recycling have their limits, large amounts of plastics continue to be landfilled.

Importantly, current gaps in literature concern the still limited characterization of efficient plastic depolymerizing enzymes which are required for the degradation of plastics into their corresponding monomers, which subsequently can be used for recycling and upcycling. In this review, we addressed the enzymatic degradation of plastic. We divide the overall approach into “upstream” and “downstream” approaches. The upstream approaches are dealing with enzymatic depolymerization of plastics while the downstream approaches are focused on upcycling and merging plastic waste management into the bioeconomy. We focus our discussion on how to identify and characterize novel enzymes, especially for the important aforementioned upstream approaches, and how to assess the suitability and efficiency of enzymatic degradation processes, providing a comprehensive workflow which so far is lacking in literature. Thus, our review is substantially different from other recent reviews in the field of microbial plastic degradation (Wang et al., 2021; Sarkar et al., 2022; Zhou et al., 2022). We refer to other recent reviews in the field of microbial plastic degradation reviewing other topics within this rapidly evolving field (Wang et al., 2021; Sarkar et al., 2022; Zhou et al., 2022).

## Diversity of Synthetic Polymers

Currently, over twenty different types of plastics are on the market, the majority developed and synthesized from petrochemical feedstocks (PlasticEurope-Association of Plastics Manufacturers, 2020). Clear differences can be observed in the biodegradability of different plastics. Important determinants for the degradability of plastics are the nature of the chemical bonds constituting the polymeric structure, and the crystallinity of the plastic. Recently, the development of biobased plastics has taken an important leap. Biobased plastics are manufactured using biological feedstocks, like starch or lactic acid. However, this does not entail that they can automatically be regarded as biodegradable. Some biobased plastics, like polylactic acid, are highly recalcitrant and hence, are difficult to degrade.

**FIGURE 1:**
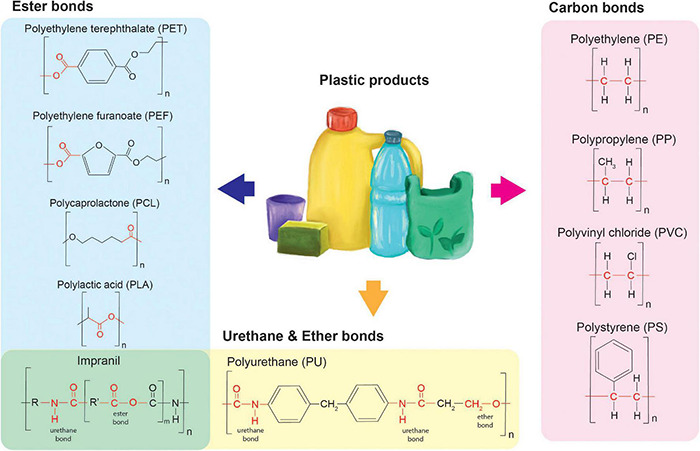
Plastics and their specific chemical bonds.

Different plastics may indeed be structurally different, however, the covalent chemical bonds connecting the constituting monomers can be similar. In Figure 1, several examples are given of different plastics containing similar chemical bonds connecting the structurally different monomers. We hypothesize that the biodegradability of these plastics correlates with the occurrence and abundance of the specific chemical bonds in natural substrates. The more likely a bond is to be present in natural substrates, the more likely it may be recognized and broken in the synthetic polymer. It is expected that the enzymes’ ability to degrade specific bonds can either be coincidental or has evolved depending on the nature of the enzyme. Determining the prevalent chemical bonds within a plastic polymer of interest provides insights into the family of enzymes needed to degrade the plastic and may give insight into possible modifications needed for these enzymes. Four types of covalent bonds are abundantly present in plastics: ester bonds, urethane bonds and carbon-carbon bonds. In addition, the crystallinity of plastics presents another important determinant for their biodegradability.

### Ester Bonds

Ester bonds are abundant in both petrochemical-derived plastics and bio-based polymers. Typical plastics containing these bonds are polyethylene terephthalate (PET), polycaprolactone (PCL), poly-lactic acid (PLA), and many more exist. Interestingly, for all of these plastics degrading enzymes have been identified. This likely correlates with the abundance of ester bonds in natural polymers such as lipid- and phenolic-based barriers present in the outer layer of plant cells. Enzymes able to degrade such natural polymers have in some cases been shown to be able to degrade synthetic compounds such as PET. A common example is presented by cutinase enzymes from *Thermobifida* spp., that degrade plant polymeric cutin and in addition have been shown to degrade PET (Ribitsch et al., 2012). Several common lipases and esterases have also been shown to be able to degrade one or more of the above-mentioned polyesters (Hajighasemi et al., 2016).

### Urethane Bonds

Urethane bonds constitute polyurethane plastics which are mostly used for long-term applications in construction and automotive, as well as in foams used e.g., in furniture (PlasticEurope-Association of Plastics Manufacturers, 2020). Urethane bonds resemble covalent peptide bonds between amino acids constituting proteins. Electron density and pulling force within these bonds are clearly different from those in ester bonds and hence, different enzymes are required for degradation (Kjeldsen and Zubarev, 2011). In addition, several enzymes such as the polyester hydrolases were shown to degrade polyurethanes (Howard et al., 1999; Russell et al., 2011; Álvarez-Barragán et al., 2016; Schmidt et al., 2017; do Canto et al., 2019).

The majority of published research on polyurethane degradation is performed on Impranil-DLN (Biffinger et al., 2015; Schmidt et al., 2017; Danso et al., 2019; Espinosa et al., 2020). Impranil is an aqueous polyester-polyurethane emulsion that is mostly used for impregnating textile (Biffinger et al., 2015). This emulsion is easily incorporated in solid growth medium and accessible for bacteria or fungi allowing for a ready to use screening method. However, it is not easily translatable to the degradation of polyurethanes like PUR (polyurethane), since Impranil also contains ester bonds (Molitor et al., 2020).

### Ether Bonds

Ether bonds are abundantly found in nature, they are present in wood cell walls (Nishimura et al., 2018), plant active molecules such as saponins (Abed and Aziz, 2019) and many more (Domínguez De María et al., 2010). Some plastics contain combinations of bonds for example PUR contains both urethane bonds as well as ether bonds (Figure 1). These foams are highly recalcitrant and difficult to degrade. So far it has been shown that a selected microbial community was able to partially degrade these polyether-polyurethane co-polymers when dispersed in water (Gaytán et al., 2020). Recently, the first case of enzymatic poly(ether urethane) foam degradation has been shown using a laccase-mediated system (Magnin et al., 2021). Butylene succinate copolymers like butylene succinate-co-diethylene glycol succinate (PBS-co-DEGS) and butylene succinate-co-butylene diglycolic acid (PBS-co-BDGA) are other examples of polymers with combined bonds since it consists of ester and ether bonds. The dispersed presence of these ether bonds in these specific co-polymers appears to improve the hydrophobicity of these plastics and hence, improves their biodegradability (Li et al., 2018).

### Carbon-Carbon Bonds

Carbon-carbon bonds are most commonly found in petrochemical-derived plastics like polystyrene (PS), polyvinyl chloride (PVC), polyethylene (PE), and polypropylene (PP). These plastics, and especially PE, are commonly used in single-use products (packaging, plastic bags, etc.) and contribute extensively to environmental pollution (PlasticEurope-Association of Plastics Manufacturers, 2020). In contrast to urethane bonds and ester bonds, carbon-carbon bonds are much more recalcitrant, and their degradation usually requires redox enzymes. These enzymes cleave the carbon-carbon bond in four steps: (1) oxidation and rearrangement of unstable oxidized intermediates; (2) collapse and rearrangement of radicals and/or cations; (3) oxygenation to obtain hydrolyzed cleavage products and finally; (4) oxygen activation requiring oxygen for substrate binding. The complex redox enzymes catalyzing these steps require metal ligands (Guengerich and Yoshimoto, 2018).

In contrast to ester bonds, only a few specific enzymes have been identified for the degradation of carbon-carbon bond polymers. Recently, initial steps were taken with the isolation of *P. putida* from the gut of polystyrene-eating super worms (Yang et al., 2020). Kim et al. (2020) isolated *Pseudomonas* sp. DSM50071 from the super worm gut and identified the enzyme responsible for polystyrene degradation. The enzyme was characterized as a serine-hydrolase oxidizing the polystyrene and thereby increasing its hydrophilicity and enabling further degradation of the polymer. For polyethylene (PE), polypropylene (PP), and polyvinyl chloride (PVC) microbial degradation has been observed. For example, PE degrading fungi have been identified using a combination of solid screening on medium containing plastic particles in combination with respirometry assays, Fourier Transform infrared (FTIR) and scanning electron microscopy (Spina et al., 2021). Several Fusarium species, *Aspergillus fructus* and *Purpureocillum lilacinum* were able to damage PE films, however, no specific enzymes were identified (Spina et al., 2021).

### Other Structural Determinants

Apart from the chemical bonds connecting the monomers, other physicochemical characteristics may affect the biodegradability of plastics. One important factor is crystallinity which affects the strength of plastic polymers as well as their degradation rate (Pantani and Sorrentino, 2013). Low crystallinity results in better degradation. PET degrading *I. sakaiensis* can degrade amorphous PET but is unable to degrade high crystallinity PET (Wallace et al., 2020). When the crystalline PET was converted to amorphous PET the bacterium again was able to degrade the compound (Wallace et al., 2020). Studies on the degradation of PBS also suggested that crystallinity may be a more important factor influencing degradation than molecular weight (Pan et al., 2018). Moreover, these authors showed that crystallinity of PBS may decrease after several hours of degradation (Pan et al., 2018). Another study showed that the crosslinking of amorphous silk domains with crystalline silk regions protects the amorphous region, making it more resistant against enzymatic degradation (Valente et al., 2020), The degradation of plastics with high crystallinity may be improved by the expansion of the enzyme’s binding pocket as shown in the case of PET (Liu et al., 2018; Kitadokoro et al., 2019) or by the pre-treatment of plastics making them less crystalline (Wallace et al., 2020).

The addition of specific biosurfactants may significantly increase or decrease the biodegradability of various plastics. Recently, it was shown that the addition of rhamnolipids increased the microbial colonization of plastics (Taghavi et al., 2021). Whether degradation was indeed increased, appeared dependent on the type of plastic. For PET and PE biodegradation decreased, whereas for PS degradation increased (Taghavi et al., 2021). This is likely to be a rhamnolipid specific feature since another study indicated increased biodegradability of LD-PE upon the addition of biosurfactants (Hussain Ali et al., 2021).

## The Search for New Plastics Degrading Microbes and Enzymes

Enzymatic recycling of plastics presents a sustainable and therefore interesting approach for several reasons. These include (*i*) no harsh conditions are required for degradation, (*ii*) costs for bulk enzyme production can be relatively low, and (*iii*) it allows for the retrieval of the monomers which can be polymerized into new plastics or upcycled into new compounds. To develop the enzymatic degradation approach, novel plastics degrading enzymes need to be discovered, characterized and improved. These enzymes can be categorized into two types: (1) plastic depolymerizing enzymes and (2) downstream processing enzymes, ensuring proper processing of the degradation products toward the desired product. For example, the PETase from *Ideonella sakaiensis* (*Is*PETase) can degrade PET into mono-(2-hydroxyethyl) terephthalic acid (MHET) and this enzyme would be categorized as a PET depolymerizing enzyme. The MHETase that degrades MHET into terephthalic acid and ethylene glycol is regarded as a downstream processing enzyme within the scope of this review (Yoshida et al., 2016). These enzymes were initially identified by Yoshida et al. (2016) and subsequently discussed in a broad spectrum of papers discussing the production, structure and mechanisms of both the PETase and the MHETase (Han et al., 2017; Liu et al., 2018; Ma et al., 2018; Almeida et al., 2019; Palm et al., 2019; Seo et al., 2019; Janatunaim and Fibriani, 2020; Wallace et al., 2020).

The search for novel complex polymer degrading enzymes started decades ago and was extended with plastics depolymerization enzymes in the late 1980s. In, Pometto et al. (1992) showed that *Streptomyces* species can produce extracellular polyethylene degrading enzymes. These enzymes can be optimized to reach a higher efficiency. In addition, *in silico* methods can be used to identify interesting homologs for further investigation. Several strategies to discover new plastic degrading enzymes are discussed in detail below. The enzymes discussed in this paragraph were summarized in Table 1.

**TABLE 1:** Comprehensive overview of enzymes discussed in this review.

Plastic	Chemical bond	Domain and phylum	Species	Method of identification	Enzyme	Enzyme class	References
PET	Ester	Bacteria—Proteobacteria	*Ideonella sakaiensis*	Screening on low-crystallinity PET films	*Is*PETase	Tannase	Yoshida et al., 2016; Liu et al., 2018
PET	Ester	Bacteria—Actinomycetota	*Thermobifida fusca*	Enzyme activity assay on 3PET	*Tf*Cut2	Cutinase	Herrero Acero et al., 2011; Ribitsch et al., 2012, 2015
PET/PEF	Ester	Bacteria—Actinomycetota	*Thermobifida cellulosilytica*	Enzyme activity assay on 3PET Enzymatic assay on PEF powder	*Tc*Cut1	Cutinase	Herrero Acero et al., 2011; Pellis et al., 2016
PET	Ester	Bacteria—Actinomycetota	*Thermobifida alba* AHK119	Screening on 3PET	Est119/*Tha-*Cut1	Cutinase/Esterase	Kitadokoro et al., 2012; Ribitsch et al., 2012
PLA	Ester	Bacteria—Proteobacteria	*Alcanivorax borkumensis*	Screening on various polymer emulsions	*ABO*2449	α/β hydrolase	Hajighasemi et al., 2016
PLA	Ester	Bacteria—Proteobacteria	*Rhodopseudomonas palustris.*	Screening on various polymer emulsions	*RPA*1511	α/β hydrolase	Hajighasemi et al., 2016
PCL	Ester	Bacteria—Proteobacteria	*Alcanivorax borkumensis*	Screening on various polymer emulsions	*ABO*2449	α/β hydrolase	Hajighasemi et al., 2016
PCL	Ester	Bacteria—Proteobacteria	*Rhodopseudomonas palustris.*	Screening on various polymer emulsions	*RPA*1511	α/β hydrolase	Hajighasemi et al., 2016
PCL	Ester	Bacteria—Actinomycetota	*Streptomyces* sp.	*In silico* search, screening on emulsified PCL	*SM*14est	Esterase	Almeida et al., 2019
PS	Carbon-Carbon	Bacteria—Proteobacteria	*Pseudomonas* sp. DSM50071	Grown on PS film	No specific enzyme	Serine hydrolase	Kim et al., 2020
LD-PE	Carbon-carbon	Bacteria—Proteobacteria	*Pseudomonas putida*	Screening on LD-PE films	No specific enzyme	Unknown	Talkad et al., 2014
LD-PE	Carbon- carbon	Fungi—Ascomycota	*Fusarium oxysporum, Fusarium falciforme and Purpureocillum lilacinum*	Initial screening on agar containing pulverized LD-PE	No specific enzyme	Unknown	Spina et al., 2021
LD-PE	Carbon-carbon	Bacteria	Marine organisms	LD-PE powder in medium	No specific enzyme	Unknown	Khandare et al., 2021

### Experimental Approaches—Environmental Screening for Plastic Degrading Enzymes

Isolating micro-organisms from environments with long-term exposure to rigid natural polymers or plastics has been a fruitful approach and led to the identification of several microbial species able to degrade plastics. This suggests that microbes may adapt toward the use of anthropogenic persistent and complex polymers.

Several bacteria displaying plastic degrading abilities have been isolated from floating debris, garbage soil, landfills, and polluted water. These include *Pseudomonas, Bacillus, Staphylococcus, Streptomyces*, and many more species (El-Shafei et al., 1998; Usha et al., 2011; Bhardwaj et al., 2013; Brunner et al., 2018). Here we describe a few interesting bacterial origins of enzymes. An example is presented by the newly identified bacterium, *Ideonella sakaiensis*, isolated from a PET bottle recycling facility, which uses PET as an energy source (Yoshida et al., 2016). The enzymes required for the depolymerization, and downstream degradation of PET were characterized and shown to act specifically and efficiently (Yoshida et al., 2016; Liu et al., 2018). Another example is presented by the Actinobacteria *Thermobifida* cutinases; these members of the esterase family can hydrolyze the primary and/or secondary ester linkage in cutin, allowing microbes to use the monomers as a carbon source. Cutinases hydrolyze ester bonds and hence, are interesting candidates to screen for the ability to degrade plastics. Since 2010 several studies indicated that cutinases, especially from *Thermobifida* species, can degrade synthetic polymers like PET (Herrero Acero et al., 2011; Ribitsch et al., 2012, 2015). The Cut1 enzyme from *Thermobifida cellulosilytica* has shown to successfully degrade PET and polyethylene furanoate powders (PEF) (Herrero Acero et al., 2011; Pellis et al., 2016).

Another interesting group of microorganisms are the fungi, they are known for their ability to degrade complex polymers such as wood, cellulose and lignin (Bugg et al., 2011; Couturier et al., 2018; Nemli et al., 2018; Zhang et al., 2016; Hou et al., 2020; Qu et al., 2021). Several fungal cutinases may also degrade PET (Suzuki et al., 2014; Sankhla et al., 2020; Anbalagan et al., 2021; Vázquez-Alcántara et al., 2021). In addition, Filamentous fungi isolated from landfill soil have been shown to degrade polyethylene. These include *Fusarium oxysporum, Fusarium falciforme*, and *Purpureocillum lilacinum* (Spina et al., 2021). Various reviews have been published providing an overview of these plastic degrading microorganisms (Pathak and Navneet, 2017; Roohi et al., 2017; Rana, 2019; Jaiswal et al., 2020; Mohanan et al., 2020; Maity et al., 2021; Priya et al., 2022). Due to the abundance of plastic degrading micro-organisms isolated from plastic polluted environments, plastic debris is considered a promising source for the isolation of potentially successful plastic degrading bacteria and fungi.

### Computational Approaches

With interesting strains or consortia identified, different approaches can be taken to pinpoint the genes encoding plastic degrading enzymes. The first approach has led to the identification of the *Is*PETase from *I. sakaiensis* in which the sequenced genome was investigated for promising open reading frames containing sequences like already known plastic degrading enzymes (Yoshida et al., 2016). The second approach includes transcriptome analysis and identification of up-and down-regulated genes in the presence and absence of plastics to reveal genes potentially important for PET degradation (Kumari et al., 2021).

#### Data Mining and *in silico* Screening for Plastic Degrading Enzymes

By performing homology searches using various databases promising enzymes can be revealed. An example is presented by the PETase-like enzyme SM14est found by searching homologs of the *Is*PETase. 30 potential enzymes were found of which one showed PCL degrading activity (Almeida et al., 2019). Surprisingly, to date, only a few papers have been published using this method to identify promising enzymes. Nevertheless, this is a promising technique to narrow down the number of bacteria and fungi for screening. It is, however, important to note that merely looking for homology at the amino acid level is not enough to find interesting enzymes. In addition, understanding and predicting the chemical characteristics of the enzymes and predicting enzyme structures are of utmost importance for the identification of promising new enzymes using *in silico* methods.

Another *in silico* source for enzyme identification is ancestral sequence reconstruction (ASR) By obtaining the possible ancestral sequence, novel enzymes can be identified via homology searches or novel proteins can be expressed by the creation of fusion proteins (Verma et al., 2019; Zitare et al., 2021). In addition, ASR provides insights into the evolutionary development of enzymes (Ruiz-Dueñas et al., 2020). So far, ASR and additional methods have been used to identify novel enzyme families and conserved structures (Voshol et al., 2017). Likewise, it can provide insight into the origin and evolution of plastics degrading enzymes. *In silico* methods indeed, have shown to provide leads aiding the search for novel enzymes and are, in our opinion, useful to gain more insights into promising enzymes.

#### *De novo* Enzymes Design

*De novo* design and development of enzymes, based on well-determined modular structures provides a possible future approach to generate a new generation of plastic degrading properties. The *de novo* design and creation of enzymes is one of the primary goals of synthetic biology and requires computational approaches. Donnelly et al. created *de novo* enzyme Syn-F4 which can hydrolyze the siderophore ferric enterobactin. The expression of Syn-F4 *in vivo* allowed a ferric enterobactin sensitive *E. coli* to survive in the presence of ferric enterobactin (Donnelly et al., 2018). New tools for the *de novo* design of enzymes are emerging rapidly. By using machine learning methods, protein prediction software is being improved drastically. The current prediction programs, however, are still lacking a user-friendly platform for end-users, resulting in limited use of these tools (Abriata and Dal Peraro, 2021). To stimulate the design of novel enzymes using computational synthetic biology approaches is dependent on the prior identification and characterization of several different plastics degrading enzymes to begin training machine learning software and providing accessible computing tools. In addition, a list of web tools for *in silico* biodegradation studies is available in a recent review (Skariyachan et al., 2022).

Another useful method is the addition of specific protein domains to create better enzymes with altered binding properties. This method has been used e.g., for the optimization of carbohydrate degrading enzymes, where carbohydrate-binding modules (CBMs) have been switched and replaced to optimize carbohydrate degrading abilities as well as thermostability and catalytic efficiency in existing enzymes (Ha et al., 2015; Meng et al., 2015). Likewise, CBMs may be useful tools to increase substrate binding of plastic degrading enzymes, since a recent paper showed that the addition of inactive polysaccharide monooxygenase *Pc*AA14A to the medium increased the efficiency of the *Is*PETase by 27 percent. This inactivated enzyme appears to bind with its CBM to the hydrophobic surface of PET, thus enabling the other enzyme to bind more efficiently (Dai et al., 2021a). Subsequent research by Dai et al. (2021b) shows that the addition of a CBM domain can increase the plastic degrading abilities of the *Is*PETase up to 86 percent, underscoring that enhancing these enzymes with extra binding domains is a promising approach for improved degradation.

## Enzyme and Strain Optimization for Plastic Degradation

Existing enzymes can be optimized using various methods to obtain a higher depolymerization efficiency and thermostability. These may be regarded as necessary requirements since plastic depolymerization is currently mostly conducted at elevated temperatures (Nakasaki et al., 2019; Xiong et al., 2020). In addition, *in silico* methods combined with experimental approaches can target specific sites for the catalytic improvement of these enzymes.

### Random Mutagenesis

Random mutagenesis and subsequent screening and selection is an effective strategy used to improve the activity of specific strains and enzymes. By exposing plastic degrading microbial strains to mutagens, such as UV, a high mutation frequency can be obtained and mutants with higher enzyme activity can be selected. If required, cycles of mutagenesis and selection can be repeated until a significantly higher efficiency is reached. Talkad et al. (2014) were able to improve the degradation of low-density (low crystallinity) polyethylene by UV and EMS treatment of *P. putida*. Via GWAS studies the SNPs responsible for the improvements can be identified. The same approach has shown to be fruitful in fungi. *Penicillium oxalicum* strain DSYD05-1 was optimized vis UV mutagenesis to increase its PCL degrading ability. The enzymes of this strain showed to have a higher PCL- degrading ability and a wider substrate specify allowing it to additionally degrade poly(β-hydroxybutyrate) (PHB) and poly(butylene succinate) (PBS) (Li et al., 2012).

### Site-Specific Mutagenesis Based on Enzyme Modeling

As a recent development, computer-aided enzyme engineering is increasingly used for the optimization of enzymes. When the crystal structure of an enzyme is known, computer-aided engineering can be used to identify important targets for improvement. Experimental approaches can then induce these changes resulting in optimized enzymes.

For example, based on the crystal structure and modeling of the *Is*PETase, site- directed mutagenesis has been performed on 15 amino acid domains in the first contact shell of the *Is*PETase, to improve its efficiency (Tournier et al., 2020). The addition of disulfide bridges to improve thermostability and mutagenesis of the residues responsible for substrate binding also drastically improved PET-depolymerization, allowing the enzyme to depolymerize 90% of the provided PET in 10 h. The optimized strain was able to degrade 16.7 g of PET per liter per hour (Tournier et al., 2020). The 90% efficiency in 10 h of the enzymatic degradation process comes close to the 98% efficiency in 8 h for chemical PET degradation (Khoonkari et al., 2015). Furthermore, the purified monomers could be used to synthesize new PET without the loss of quality of the material (Tournier et al., 2020).

In addition, Liu et al. (2018) enzyme modeling and experimental data provided insight into the different binding pockets of the PETase from *I. sakaiensis* and of a spectrum of cutinases. Likewise, Kitadokoro et al. (2019) identified the binding pocket residues involved in substrate interaction of a cutinase isolated from *Thermobifida* and how this differs from the *Is*PETase. After comparison with a.o., *Thf*42_Cut1 it was concluded that the success of the *Is*PETase enzyme is caused by the structural features of its binding pocket. Compared to other enzymes, this PETase has a relatively shallow and broad surface, allowing the enzyme to bind to aggregated PET molecules whereas other cutinases are mostly only able to hydrolyze linear PET molecules (Liu et al., 2018).

A similar approach was taken with the *Tf*Cut2 from *Thermobifida fusca*. Furukawa et al. (2019) managed to improve the PET degrading abilities of *Tf*Cut2 by identifying important residues via computer modeling and applying site-directed mutagenesis on the identified substrates thereby increasing the degradation rate of the PET film 12.7-fold.

In another study, the *Thc*_Cut2 cutinase was shown to be significantly less efficient than its close relative *Thc*_Cut1. Previously, Herrero Acero showed that differences in surface properties of these enzymes are responsible for the difference in efficiency. In one of the first site-specific mutagenesis studies on *Thc*_Cut2, Arg29 was mutated into Asn and Ala30 into Val. These changes resulted in significantly higher specific activity on a PET surface (Herrero Acero et al., 2013; Ribitsch et al., 2015). In the future, site-directed mutagenesis combined with the addition of substrate-binding domains (3.3) is promising to further improve the efficiency of plastics degrading.

### Adaptive Laboratory Evolution to Improve the Activity of Plastic Degrading Strains

Adaptive laboratory evolution (ALE) is a powerful approach to improve or create certain phenotypes in microbial strains by provoking and stimulating evolutionary adaptation processes (Lee and Kim, 2020). In combination with omics-approaches to characterize the mutations invoked, ALE is a sophisticated and potent strain engineering tool for inducing mutations to improve metabolic pathways and enzymes for rapid growth on a variety of carbon sources and stress tolerances. Various examples of ALE for improved utilization of plastics monomers have emerged, which are important to build plastic-degrading or -upcycling cell factories.

Genome sequencing of *Pseudomonas pseudoalcaligenes* CECT 5344 revealed that this strain could potentially utilize furfurals (monomers of the biobased plastic polyethylene 2,5-furandicarboxylate or PEF), furfuryl alcohol, furfural and furoic acid as carbon sources. However, a long lag period lasting for several days was observed during its growth on furfurals. Growth on furfurals was improved using ALE, following which the adapted strain no longer exhibited any prolonged lag phases (Igeño et al., 2019). Sequencing of this strain revealed a point mutation in an AraC family activator locus (BN5_2307) at the HTH region of the protein (L261R) to be responsible for this improvement by generating an active regulator for the *hmfABCDE* gene cluster located upstream of this gene.

In another example, Li et al. (2019) successfully isolated ALE-derived mutants of *P. putida* KT2440 able to utilize ethylene glycol, a monomeric component of PET besides terephthalate, as its carbon source. Genomic analysis of these mutants revealed missense mutations and a 15 bp deletion on PP_4283 encoding for *gclR*, a transcriptional repressor to the glyoxylate carboligase pathway. In addition, secondary mutations were also found in a transcriptional regulator encoded by PP_2046 and a porin encoded by PP_2662 which further improved the growth of ALE-derived *P. putida* KT2440 on ethylene glycol. These secondary mutations likely maintain flux balances through the initial oxidation of ethylene glycol to glyoxylate.

Although current ALE approaches are mainly focused on the efficient utilization of plastics monomers, ALE has a great potential to evolve and improve plastics depolymerization enzymes. Several members of promiscuous enzyme classes, like esterase, lipase and cutinase, have been identified to be able to depolymerize PET and PLA, although with low specificity and turn-over (Kawai et al., 2019). ALE or directed evolution constitutes a promising approach to acquire new or improved enzymatic activity from promiscuous enzyme classes to develop novel plastic depolymerizing enzymes.

## The Challenges of Experimental Design and Set-Up

The diversity of methods available for the identification of micro-organisms and enzymes with plastic depolymerizing abilities complicates the trustworthiness and translatability of the experiments. In any case, the experimental setup is highly determinant to increase the chances of success.

Clearance assays are frequently used in screening for enzyme activities. These rely on a turbid plastic-containing medium. If the plastic in the medium is degraded, a clear halo will occur. Screening for halo formation is a relatively quick method that yields binomial results. Two approaches are frequently used to incorporate plastics in the medium which include emulsification and the addition of semi-water dissolvable plastics. During emulsification, plastics are dissolved in an organic solvent, such as dichloromethane, and mixed with surfactants and growth medium. Subsequently, the solvent is evaporated, resulting in small droplets of plastic in the medium (Pranamuda et al., 1997). This method is used for plastics like polylactic acid, polycaprolactone, and polystyrene. We experienced that emulsification is not an easy-going method, difficult to reproduce and a challenge in keeping the final dispersion stable. Another approach is the addition of plastic granules or pellets to the medium and screen for clearance (Khandare et al., 2021; Spina et al., 2021).

An easier approach is the addition of plastics that are partially dissolvable or plastic simulators such as the aquatic dispersion Impranil-DLN, which is used for the screening of polyester and polyurethane degradation (Molitor et al., 2020). Similarly, bis-(2-hydroxyethyl) terephthalate (BHET) is soluble in water and can be used as a PET-mimicking substrate (Palm et al., 2019). Model compounds are very useful to show e.g., polyester degrading activity but are only partially translatable to the degradation of the actual polymer of interest (Table 2; Ribitsch et al., 2011; Almeida et al., 2019; Palm et al., 2019; Molitor et al., 2020). Therefore, one must be aware that results are easily over-interpreted and plastic degrading features may be overstated. However, plate clearance assays are quick, high throughput and important screens for the initial identification of interesting organisms.

**TABLE 2:** Overview of methods for screening and identification of plastic degradation.

Plastic	Model polymer	Method of incorporation	References
PET	PET	Emulsification	Charnock, 2021
PET	3PET, 2PET, BHET	Dissolving	Herrero Acero et al., 2011; Ma et al., 2018; Palm et al., 2019
PLA	PLA	Emulsification	Pranamuda et al., 1997
PLA	PLA	Film on plate	Kim et al., 2017
PLA	PLA	Spray	Shin et al., 2021
PCL	PCL	Emulsification	Almeida et al., 2019
PUR	Impranil-DLN	Dissolving	Molitor et al., 2020
HD-PE	HD-PE	Films	Awasthi et al., 2017
LD-PE	LD-PE powder	Mixing with medium	Khandare et al., 2021; Spina et al., 2021
PS	PS	Emulsification	Tang et al., 2017
PS	PS	Spray	Shin et al., 2021

Measuring the weight loss of plastics is another method for the identification of plastic degradation. Plastic particles or films are incubated in the presence of microorganisms and regularly weighed to monitor the possible decline in weight. Major drawbacks of this method are timespan and weight fluctuations (Li et al., 2008; Danso et al., 2019; Przemieniecki et al., 2020). It may take weeks to months to conduct the experiments and weight differences may be caused due to fragmentation by other external or internal factors such as the attachment of microbes to the plastic which is also measured. A better method to examine plastic degradation is provided via respirometry assays, specifically while the plastic is being used as sole carbon. This method is based on measuring carbon dioxide production upon degradation of the polymers, using pH indicators and carbon dioxide calibration (Yang et al., 2020; Spina et al., 2021). This method provides more reliable results than weight loss assays since it is less influenced by other external factors such as the fragmentation of the plastics. Plastics degradation may be examined by combining respirometry assays with microscopy, testing the plastic’s physical characteristics and enzymatic activity assays (Janczak et al., 2018; Tournier et al., 2020; Yang et al., 2020; Spina et al., 2021). Interactions of the microorganism with the plastic surface can be visualized using scanning electron microscopy, this does not show enzymatic activity but can provide clues about the physical interaction of the microorganism of interest and the plastics. In addition, Liquid Chromatography-Mass Spectrometry (LC-MS) is a powerful tool for the detection of polymer degradation products and characterization of the degradation pattern (Hajighasemi et al., 2016). An excellent example is the research of Hajighasemi et al. (2016), in this study purified enzymes were used to degrade PLA resulting in a clear chemical degradation pattern.

Another method to observe chemical degradation is FTIR spectroscopy, based on the (changing) absorbance or transmission of specific wavelengths by specific (altered) chemical groups (Sandt et al., 2021). Several studies used FTIR to evaluate microbial PE, PET and polystyrene degradation (Ioakeimidis et al., 2016; Ojha et al., 2017; Canopoli et al., 2020; Yang et al., 2020; Spina et al., 2021). A recently published short communication by Sandt et al. (2021) discusses the use and misuse of FTIR in relation to plastic degradation. Importantly, most studies reported to date are focused on strains displaying plastic depolymerizing abilities instead of the specific enzyme responsible. If enzymes are identified, efficiency is not easily comparable to other enzymes. Herrero Acero et al. (2011) performed several enzymatic assays, PET hydrolysis tests, modeling and docking analyses to be able to compare the Thf42_Cut1 enzyme from *Thermobifida fusca* with two other, previously identified, PET degrading cutinases: Thc_Cut1 and Thc_Cut2 derived from *Thermobifida cellulosilytica* DSM44535. By expressing and purifying these three enzymes in *E. coli* the kinetic properties on PET could be compared. They were able to model the hydrolysis of PET and determine that Thc_Cut1 was most efficient in PET degradation (Herrero Acero et al., 2011). Other methods may include examining enzyme activity on model substrates or actual polymers, as well as examining degradation using chemical analysis or microscopy (Hajighasemi et al., 2016; Austin et al., 2018; Magnin et al., 2019). An interesting approach is the use of dye-containing plastic films, whereupon enzymatic degradation of the films the dye is being released, causing the appearance of a blue color (Shinozaki et al., 2013). For enzymatic assays to work optimally the suitable conditions for enzyme activity should be determined (Jain et al., 2020).

As discussed by Arnling Bååth et al. (2020), the kinetics of these plastic degrading enzymes is not well understood and there is no framework present to analyze these reactions and therefore score the efficiency of plastic degrading enzymes. There is a need for better substrates to quantify the plastic degrading enzyme activity. Arnling Bååth et al. (2020) showed that the addition of putative attack sites on the surface of PET allowed better comparison of the activity of various enzymes. A faster method is the use of spectrophotometric absorbance to analyze the enzymatic degradation kinetics. With bulk absorbance assays insight may be obtained into the enzyme reaction kinetics and efficiency (Zhong-Johnson et al., 2021). If this method could be used as a standardized method for the comparison of plastic degrading enzymes, such comparison would be simplified drastically.

## Toward a Comprehensive Workflow for Plastic Degrading Enzyme Identification

Not only is it important to follow the currently used methods, but it is also important to establish new strategies for finding and optimizing plastic degrading species and enzymes. This will allow to fill current gaps in the identification of plastic degrading enzymes and create efficient pipelines for the identification of such novel enzymes.

**FIGURE 2:**
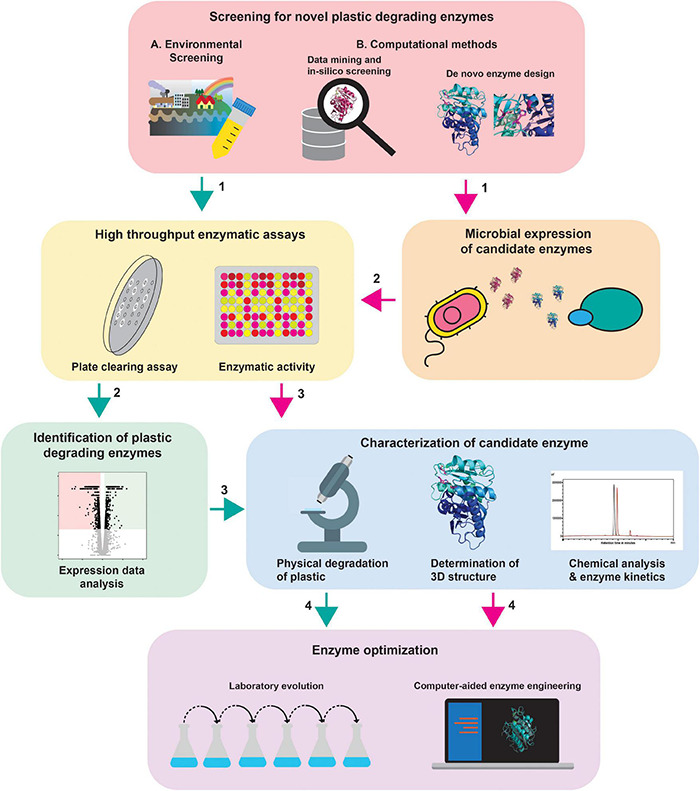
Comprehensive workflow for the identification of plastic degrading enzymes. New plastic degrading enzymes can be found by **(A)** environmental screening of interesting strains (green arrows) and **(B)** the computational methods (magenta arrows). In the case of computational methods, the enzymes need to be expressed in a suitable expression system (2) before high throughput enzyme assays can be performed. Via high throughput enzymatic assays, active strains/enzymes can be pinpointed. For the strains isolated via environmental screening, the specific enzymes need to be identified (2). The enzymes can then be further characterized to identify potential targets for enzyme optimization approaches. The protein depicted in this figure is based on PDB ID 6ANE (Fecker et al., 2018).

The diversity of methods complicates the comparison between results. Therefore, we would like to propose a workflow that would make outcomes more comparable and would result in more experimental support for future experiments (Figure 2).

As a quick and reliable method for screening organisms for plastic degrading activities, we suggest using the clearance assays since this method is also suitable for high throughput screening. To screen efficiently, optimal screening conditions need to be determined and possible inducers for enzyme expression should be identified. These screens can be performed using model polymers, or by emulsification, incorporation into the medium or spraying of the original polymers (Table 2).

The most favorable strains can be grown in liquid culture containing the above mentioned model plastics, or the actual plastic polymer itself in powder, film, particle or even cube form (Chua et al., 2013; Sepperumal et al., 2013; Pellis et al., 2016; Weinberger et al., 2017; Magnin et al., 2021). By exposing microorganisms to plastic particles in liquid culture, the degradation can be examined providing more insights into the natural degradation processes. Samples from these flasks can be used for several approaches; (1) microscopical analysis, the physical interaction between de microorganism and the plastic can be examined by SEM by making high-resolution images of the interactions between the organism of interest and plastic. A drawback is that only the macrostructure of the film or particle can be observed since it is only a visual technique. (2) Chemical analysis, for example, LC-MS-based analyses to observe and identify the non-metabolized products. In addition, FTIR can detect and indicate the oxidation of the plastics and the creation of new chemical bonds during this process (Sandt et al., 2021). Identification of complete chemical degradation can prove difficult when the degradation products are being metabolized. This can be visualized using respirometry monitoring carbon dioxide release and provide proof for the use of plastic as a carbon source (Yang et al., 2020; Spina et al., 2021). Another, however, more sophisticated option would be the use of a cell-free system to observe and investigate actual degradation by specific enzymes.

(3) Genomics, transcriptomics and/or proteomics can be used to identify the responsible enzyme(s) and optimized using ALE or site-directed mutagenesis approaches. Parts of this workflow can be used depending on the starting point of the study (Figure 2).

## The Future of Plastic Depolymerization—Biorecycling and Bio-Upcycling

When plastic degrading strains or enzymes are identified, characterized, and improved, the next step would be to apply the strains or enzyme (cocktails) for the degradation of plastics. We identified two main fields for the application of these strains or enzymes namely: (1) biorecycling and bio-upcycling, (2) bulk degradation and bioremediation. Depending on the application a thoughtful future approach is suggested.

Biorecycling and bio-upcycling are promising solutions to replace the current mechanical recycling methods since less product and quality is lost. Purified enzymes or enzyme cocktails can be used to catalyze the depolymerization process. Some advantages of a cell-free system are the mild reaction conditions and degradation under conditions unsuitable for bacterial or fungal cultures. The major challenge to overcome, still, is the handling of combinations of different plastics since no enzyme is expected to be active on all plastics. Large scale degradation of plastic mixtures can be achieved using enzyme cocktails. Such mixtures can be used to degrade mixes of different polymers or one specific complex polymer consisting of different chemical bonds. Enzyme cocktails are already used in industry for the degradation of natural complex compounds, such as lignocellulose (Lopes et al., 2018). Currently, projects are starting up researching the use of enzyme cocktails for plastic degradation (Ballerstedt et al., 2021).

Another promising method is presented by the industrial degradation of plastics by microbial consortia (Skariyachan et al., 2021). Microbial consortia have shown to degrade PE, PP, and other recalcitrant polymers (Skariyachan et al., 2017; Kang et al., 2020; Blair et al., 2021). These can be carefully structured consortia (Negi et al., 2009; Kang et al., 2020) or specifically isolated consortia from waste (Skariyachan et al., 2016, 2018). No economic models have so far been published about the feasibility of such approaches on an industrial scale. However, if plastic can be used as a sole carbon source for these communities, the use of microbial consortia for plastic degradation would be a cost-efficient approach once the upscaling is successful.

To achieve large scale degradation of these plastics using a cell-free system, the efficiency of the plastic degrading enzymes must be increased, and an efficient waste retrieval system must be established. PET would be an ideal candidate plastic for the establishment of a large-scale enzymatic recycling trial. Many countries already have an established PET retrieval system in place meaning enough PET waste can be supplied. The availability of relatively pure PET waste and PET degrading enzymes to convert PET into its monomers allows efficient recycling. These monomers can then be purified and repolymerized into new plastics (Tournier et al., 2020). The monomers can also be upcycled toward more valuable compounds. As discussed in the bowtie model by Tiso et al. (2021), bacterial metabolism can be adapted to convert plastic imputes (monomers, oligomers) into a wide variety of compounds such as aromatics, organic alcohols and more. The promise of this has already been shown by the conversion of terephthalic acid to vanillin, coumarin and catechol (Kim et al., 2019; Sadler and Wallace, 2021). The review of Tiso et al. (2021) discusses the promise of engineered microorganisms for the processing of these plastics and the promise of plastic monomers as substitutes for current petrochemical-based materials extensively. The adjustments of these metabolic pathways were extensively described showing the possibilities to further engineer these pathways allowing for microbial upcycling of plastics (Tiso et al., 2021).

There are some challenges to overcome, especially concern plastic depolymerizing enzymes. The main demerits, concerning plastic degrading enzymes, will so far remain enzyme efficiency, thermostability and the degradation of highly crystalline plastics. These aspects need to be dramatically improved to be able to degrade, for example, high-crystallinity PET used for the manufacture of plastic bottles (Yoshida et al., 2016; Kawai et al., 2019; Kitadokoro et al., 2019). Hence, the identification and optimization of (novel) enzymes promise to yield enzymes efficient enough to degrade high crystallinity plastics. Additionally, thermostability of enzymes can be improved using enzyme modeling combined with site-specific mutagenesis (Liu et al., 2018; Tournier et al., 2020). Another option would be the optimization of the degradation conditions, to allow for more efficient degradation. This approach has been taken regarding the microbial production of plastics and could also be an important aspect for its degradation (Thapa et al., 2018; Sabapathy et al., 2019; Mohammed et al., 2020).

Another important aspect that must be considered is where these methods fit within the global development of a sustainable society and circular economy. Implementation of circularity entails that optimally no plastics would be incinerated or lost but all would be reused or recycled (Bucknall, 2020). So far, the circular economy has been implemented only partially since more time and money is needed to create the necessary infrastructure. The requirement to close the loops is highly dependent on the type of plastic; for each type, another approach would be needed (Eriksen et al., 2019). Additionally, several chemical plastic recycling methods have been shown not to be economically viable (Bucknall, 2020). An extensive comparison between all plastic recycling methods has recently been described by Lee and Liew (2021). Currently, a vast knowledge gap still exists considering the economic feasibility of enzymatic and/or microbial recycling in large scale (industrial) applications. Economical models must be established to further investigate the financial feasibility of these methods (Lee and Liew, 2021).

## Concluding Remarks

Promising steps have been taken in the biorecycling, upcycling, and biodegradation of synthetic polymers. By focusing on the degradation of specific chemical bonds instead of degradation of specific plastics, the search can be targeted to more all-round or generalist plastic degrading enzymes. We hypothesize that the abundance of specific bonds in natural polymers makes degradation of these bonds in synthetic polymers more likely. Further, several strategies to find plastic degrading organisms were mentioned to provide an overview of promising niches or environments to find possible plastic degrading organisms. The discovered strains and enzymes can be optimized using various techniques.

Experimental screening for plastic degradation can be complicated because plastics are intrinsically hard to degrade and difficult to disperse in the medium. Since experiments with plastics are not very straightforward, many different methods are used in literature. To compare studies with each other a standardized workflow was set up and described in this paper to provide guidelines toward the discovery and optimization of plastic degrading enzymes. This workflow can be used to find and optimize plastic degrading enzymes for biorecycling, bio-upcycling, bulk degradation, or bioremediation.

The utilization of enzymatic cocktails can be an attractive solution considering plastic mixtures, either mixed in the waste stream or products containing several plastics. Therefore, future research should be directed toward the optimization of enzymatic cocktails for plastic degradation. We are convinced that using the microbial diversity, enzyme optimization approaches and optimization methods to produce these enzymes cost-effectively will provide a sustainable method for enzymatic plastic degradation and recycling.

## Author Contributions

J-AV wrote the majority of the main text of the manuscript. HK contributed to the writing of the manuscript and designed the figures. J-AV, HK, AR, and JW did the conceptualization, reviewing, and editing. All authors contributed to the article and approved the submitted version.

## Conflict of Interest

The authors declare that the research was conducted in the absence of any commercial or financial relationships that could be construed as a potential conflict of interest.

## Publisher’s Note

All claims expressed in this article are solely those of the authors and do not necessarily represent those of their affiliated organizations, or those of the publisher, the editors and the reviewers. Any product that may be evaluated in this article, or claim that may be made by its manufacturer, is not guaranteed or endorsed by the publisher.
